# Distributional patterns of subchondral bone density and histopathological features of the first tarsometatarsal joint in hallux valgus feet

**DOI:** 10.1186/s12891-022-05523-2

**Published:** 2022-06-14

**Authors:** Yasunari Ikuta, Tomoyuki Nakasa, Junichi Sumii, Akinori Nekomoto, Nobuo Adachi

**Affiliations:** 1 Department of Orthopaedic Surgery, Graduate School of Biomedical and Health Sciences, Hiroshima University, Kasumi 1-2-3, Minami-ku, Hiroshima, 734-8551 Japan; 2 Sports Medical Center, Hiroshima University Hospital, Hiroshima, Japan; 3 Medical Center for Translational and Clinical Research, Hiroshima University Hospital, Hiroshima, Japan

**Keywords:** Arthrodesis, Hallux valgus, Osteoarthritis, Subchondral bone density, Tarsometatarsal

## Abstract

**Background:**

Hypermobility of the first tarsometatarsal (TMT) joint is frequently identified in patients with hallux valgus (HV); however, its association with the development of osteoarthritis in the first TMT joint in such patients remains unknown. The purpose of this study was to clarify the distribution of subchondral bone density of the first TMT joint via computed tomography (CT) using Hounsfield units (HU).

**Methods:**

Patients were divided into three groups: the osteotomy (20 feet; 20 women, mean age: 61.8 years), arthrodesis (23 feet; two men, 21 women, 71.2 years), and control group (patients without HV deformity who had undergone CT scans of the foot; 13 feet; seven men, six women, 29.7 years). The HU ratios were calculated, which were defined as the HU value of each subdivision of the subarticular spongiosa of the first TMT joint [dorsomedial (DM), dorsolateral (DL), plantomedial (PM), and plantolateral (PL)] divided by the HU values of the entire joint surface. The ratios for the osteotomy, arthrodesis, and control groups were compared. The degradation of the articular cartilage in the first TMT joint was histologically graded in the arthrodesis group. Tukey–Kramer multiple comparison analysis was conducted to compare the HU ratios among the three groups, and the histological grade in each subdivision.

**Results:**

The arthrodesis group demonstrated high HU ratios in the DM area of the medial cuneiform, and significantly lower HU ratios in the PL area of the first metatarsal. Lower HU ratios in the DL area were observed in both the osteotomy and the arthrodesis group when compared to that in the medial cuneiform of the control group. The histological evaluation indicated nearly normal articular cartilage for all subdivided areas in both the medial cuneiform and the first metatarsal in patients with severe HV.

**Conclusions:**

Although high subchondral bone density was identified in the DM area of the medial cuneiform in severe HV, only mild degradation was histologically observed in the articular cartilage of the first TMT joint. Our findings suggest that the indications for arthrodesis of the first TMT should be reconsidered based on the severity of the degenerative changes in the first TMT joint.

## Background

Hallux valgus (HV) is a common pathological deformity of the foot, with a predilection toward old age and female sex and a standardized prevalence of 28.4% among the primary care population aged 40–80 years. The prevalence of HV was reported to be higher in women (38%) than in men (21%) [1]. Anatomical and biomechanical factors—metatarsus primus varus, metatarsal morphology, ligamentous laxity, and the first ray hypermobility—play an important role in the pathogenesis of HV [2, 3]. The first ray, which includes the first tarsometatarsal (TMT) joint, serves an important biomechanical function within the foot [4], while clinical hypermobility of the first TMT joint was observed in 96% of patients with recurrent HV, with 52% of these patients exhibited radiographic evidence of first TMT joint instability [5]. Joint instability is highly associated with the development of osteoarthritis (OA) in the knee, ankle, and shoulder joints owing to abnormal loading due to changes in contact stress, distribution, and directional gradients.[6] Although the population prevalence of symptomatic radiographic OA in the first TMT joint is 3.9% among adults aged 50 and older [7], the correlation between the incidence of OA and hypermobility or instability in the first TMT joint—the severity of HV—is not fully understood.

Subchondral bone pathology leads to cartilage degeneration by altering the biomechanical force distribution across the joint cartilage, while an increase in the subchondral bone mineral density is associated with the structural progression of OA [8, 9]. The pattern of distribution of subchondral bone density reflects long-term mechanical stress in the joint [10]. Recently, studies have reported the use of several methods, such as plantar pressure measurements and finite element foot models to evaluate the biomechanical characteristics of foot in patients with HV [11, 12]. In addition, high-resolution peripheral quantitative computed tomography (HR-pQCT) can be performed to assess bone microstructure; however, it is not used widely. Alternatively, conventional computed tomography (CT) images can be used to evaluate osteopenia and osteoporosis via Hounsfield unit (HU) measurements based on a defined scale ranging from 0 for water to -1000 for air (representing the density of tissue). HU values are standardized linear attenuation coefficients for tissue [13]. Studies have reported the use of HU measurements to determine the subchondral bone density, in which specific stress distributional patterns of the medial gutter in the talocrural joint and the lateral aspect of the subtalar joints were studied in patients with chronic lateral ankle instability [14, 15]. Previous studies on bone density analysis have revealed that the distal-dorsolateral area is the densest portion of the medial cuneiform [16]. However, the region of interest was set as the main body of the medial cuneiform, which differs from the subchondral bone plate and subarticular spongiosa; therefore, we focused on subchondral bone density as a predisposing factor for OA.

This study was aimed to investigate the difference in the subchondral bone density of the first TMT joint between normal feet and those with HV, and the associated histopathological features in severe HV with metatarsus primus varus. We hypothesized that the distributional pattern of subchondral bone density via HU measurements can help in detecting OA in the first TMT joint.

## Materials and methods

This study was approved by the Ethics Committee of Hiroshima University Hospital. All patients provided informed consent. Among patients who underwent surgical treatment for HV deformity at Hiroshima University Hospital between February 2019 and October 2020, we retrospectively investigated patients with preoperative CT images of the foot that allowed for the measurement of HU (Table 1).

**Table 1:** Demographic data of the control, osteotomy, and arthrodesis groups

		**Control** **(*n* = 13)**	**Hallux valgus** **Osteotomy** **(*n* = 20)**	**Hallux valgus** **Arthrodesis (*n* = 23)**	*P* value
Age (years)		29.7 (19–46)	61.8 (28–79)	71.2 (64–79)	< 0.01*
Sex	Male	7	0	2	< 0.01**
	Female	6	20	21	
Side	Left	5	10	10	0.66**
	Right	8	11	13	

^*^One-way ANOVA

^**^Chi-squared test

Age is presented as mean (range)

### Participants

#### Osteotomy group

Distal (Mitchell or chevron procedure) and proximal (modified crescentic or closing wedge osteotomy) first metatarsal osteotomies were performed in patients with mild or moderate HV without lesser toe deformities. Twenty patients (20 women; mean age, 61.8 years; range, 28–79 years) were included in the osteotomy group.

#### Arthrodesis group

Modified Lapidus arthrodesis was employed both in patients with severe HV deformity and those with moderate deformity combined with metatarsophalangeal joint dislocation of the lesser toe, comprising 23 feet in total (two men, 18 women; mean age, 71.2 years; range, 64–79). Out of these, three patients underwent bilateral foot surgery, and five were diagnosed with rheumatoid arthritis; additional procedures were performed for the accompanying lesser toe deformities. Proximal oblique shortening, Weil, and modified Coughlin osteotomies were performed in 12 (second metatarsal, 12; third metatarsal, four; fourth metatarsal, one), two, and five cases, respectively.

#### Control group

The control group included patients without HV deformity who underwent both weight-bearing plain radiography and CT of the foot due to other foot and ankle disorders. Patients with HV, determined by an HV angle (HVA) equal to or greater than 20° on the weight-bearing dorsoplantar view, were excluded from the control group. One patient underwent a CT scan of both feet. Consequently, 12 patients (representing 13 feet; seven men, six women; mean age, 29.7 years; range, 19–46) were included in the control group. Diagnostic data of the control group is as follows: osteochondral lesion of the talus in three feet; posterior ankle impingement syndrome in three; a bone tumor in one; calcaneal nonunion in one; lateral ankle ligament injury in one; loose body in the ankle in one; os subfibulare in one; talocalcaneal coalition in one; and tarsal bone fracture in one.

### Image-based assessment

**Fig. 1:**
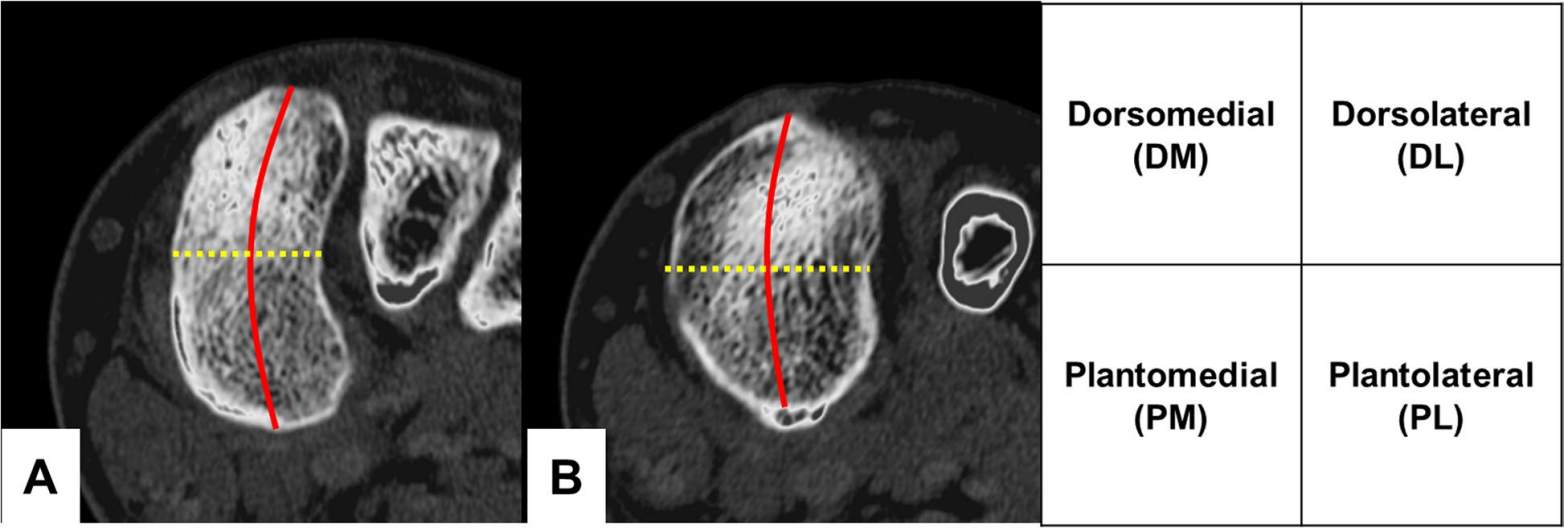
Four subdivided areas of the first tarsometatarsal joint in the medial cuneiform (**A**) and the first metatarsal (**B**) on axial CT images. Red line: line that connects the midpoint of the mediolateral width; Yellow dotted line: bisection line of the dorsoplantar height

Radiographic parameters of HV deformities were measured on preoperative weight-bearing dorsoplantar radiographs of the foot, including the HVA, as well as the intermetatarsal angle (IMA) between the first and second (M1-2), and the first and fifth (M1-5) metatarsal bones. Preoperative CT were performed using a multidetector-row CT scanner (LightSpeed QX/I; GE Healthcare, Chicago, IL, USA), and its findings were retrospectively reviewed. The calibration of the CT scanner to guarantee standardized image quality was conducted monthly. The imaging parameters included a 512 × 512 matrix, 0-degree gantry tilt, 1.25-mm prospective slice thickness, 120 kV (peak) tube voltage, and 120–200 mA tube current. Two-dimensional images were reconstructed with a 25-cm field of volume, 1.25-mm retrospective slice thickness, and 0.63-mm overlap. Axial slices parallel to the first TMT joint were obtained from the CT data. Each axial slice below the subchondral bone plate of the medial cuneiform and first metatarsal was divided into four areas: the dorsomedial (DM), dorsolateral (DL), plantomedial (PM), and plantolateral (PL) areas. The dorsoplantar and mediolateral areas were demarcated by the bisector of the dorsoplantar height and the line that connects the midpoint of the mediolateral width, respectively (Fig. 1). The average HU values of subarticular spongiosa except for the cortical shell in the whole joint surface as well as the subdivided areas were determined by delineating the region of interest in both the medial cuneiform and first metatarsal using ShadeQuest/ViewR-DG V1.26 (Fujifilm Medical Co., Ltd., Tokyo, Japan). The HU ratio was calculated by dividing the HU values of each subdivision with the HU values of the whole joint surface. These ratios were then compared among the osteotomy, arthrodesis, and control groups.

### Histopathological assessment

The articular cartilages and the subchondral bones in the first TMT joint were intraoperatively harvested and resected during modified Lapidus arthrodesis. All tissues were embedded in paraffin after fixation in 10% formalin neutral buffer solution (Wako Pure Chemical Industries, Ltd., Osaka, Japan), and decalcified in 0.5 mol/L ethylenediaminetetraacetic acid disodium salt solution (Nacalai Tesque Inc., Kyoto, Japan). The osteochondral tissues were sectioned (4.5 μm) and stained with Safranin-O/fast green, and the articular cartilage was histologically graded according to the Osteoarthritis Research Society International (OARSI) osteoarthritis cartilage histopathology assessment system [17]. The grades were then compared among the subdivided areas, such as the DM, DL, PM, and PL areas, similar to that in the CT assessment.

### Statistical analysis

Patient age and HU ratios of the subarticular spongiosa in each subdivided area were compared among the groups using one-way analysis of variance (ANOVA) and Tukey–Kramer multiple comparison analysis as results of the normality test (Shapiro–Wilk test, *P* > 0.05) and homogeneity of variance test (Levene’s test, *P* > 0.05), with *P* values < 0.05 considered significant. The Chi-square test was used to compare the sex and the affected side among the three groups, while the OARSI grades of each subdivided area in both the medial cuneiform and first metatarsal were compared using one-way ANOVA and Tukey–Kramer multiple comparison analysis. The OARSI grades were compared between patients with and without rheumatoid arthritis using Welch’s *t*-test. Statistical analyses were performed using IBM SPSS Statistics for Windows, Version 27.0 (IBM, Armonk, NY, USA).

## Results

Radiographically significant differences were identified in the HVA and IMA among the three groups. No HV deformities were observed in the control group (mean HVA, 12.1; IMA, 9.4), whereas moderate deformity was noted in the osteotomy group (mean HVA, 32.7; IMA, 14.8) and severe deformity in the arthrodesis group (mean HVA, 45.4; IMA, 18.9) (Table 2).

**Table 2:** Radiographic parameters of hallux valgus

	**Control** **(*n* = 13)**	**Hallux valgus**		*P* value		
		**Osteotomy (*n* = 20)**	**Arthrodesis (*n* = 23)**	Control versus Osteotomy	Control versus Arthrodesis	Osteotomy versus Arthrodesis
**Hallux valgus angle (deg)**	12.1 (8.8–15.3)	32.7 (21.8–42.4)	45.4 (41.8–49.0)	< 0.01	< 0.01	< 0.01
**Intermetatarsal angle (M1-2) (deg)**	9.4 (8.2–10.6)	14.8 (12.2–19.8)	18.9 (17.9–19.9)	< 0.01	< 0.01	< 0.01
**Intermetatarsal angle (M1-5) (deg)**	27.6 (26.0–29.2)	35.9 (31.5–41.3)	37.5 (35.5–39.6)	< 0.01	< 0.01	0.49

All parameters were statistically analyzed using Tukey–Kramer multiple comparison analysis. Each parameter is shown as the mean (95% confidence interval)

*Abbreviation: M* metatarsal bone

**Fig. 2:**
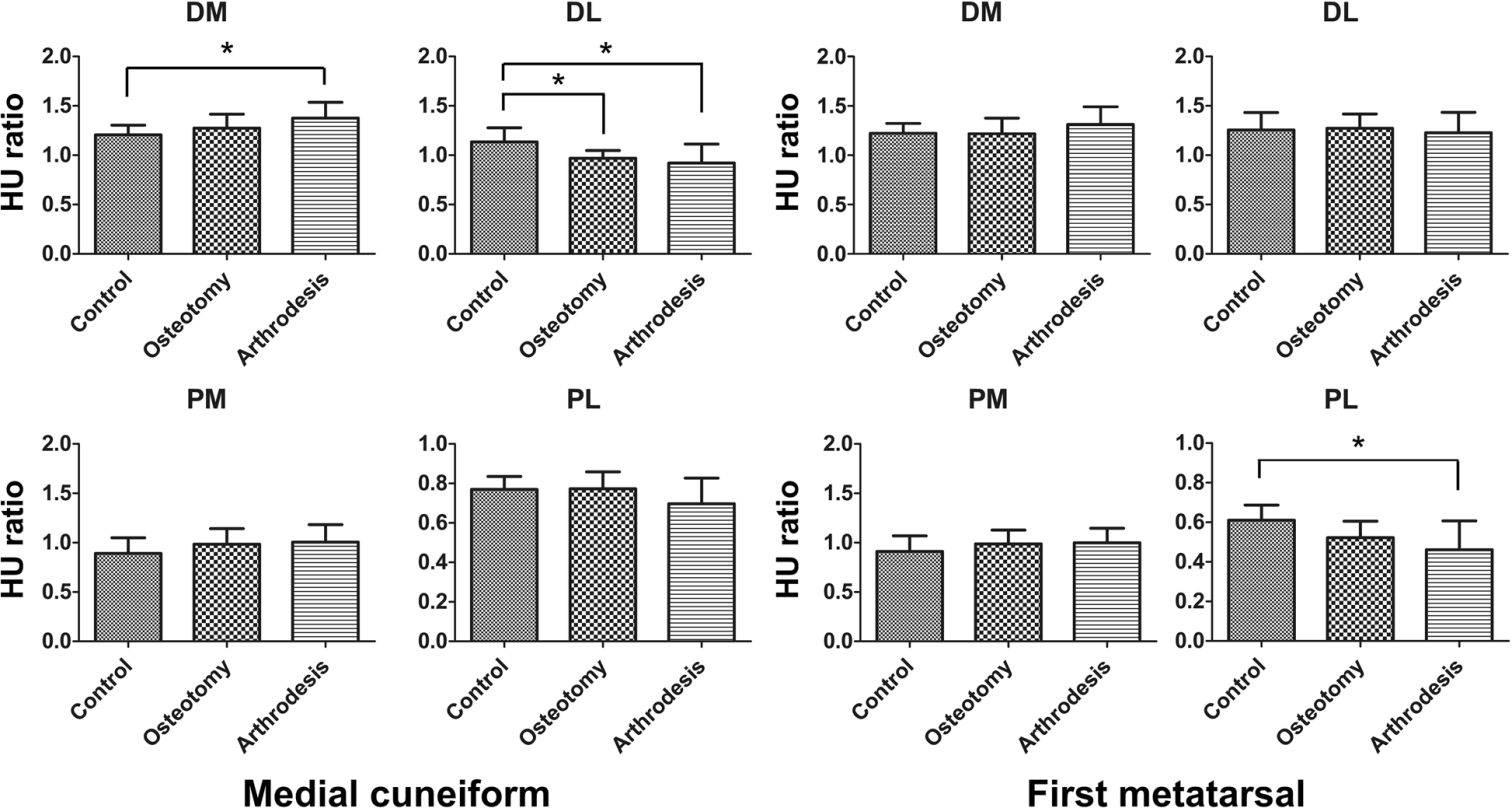
HU ratios of subdivided areas of the medial cuneiform and first metatarsal in the control, osteotomy, and arthrodesis groups. HU ratios are presented as mean ± standard deviation. * *P* < 0.05

The arthrodesis group exhibited significantly higher HU ratios in the DM area of the medial cuneiform than the control group (*P* = 0.0027). Additionally, in the DL area, lower HU ratios were noted in the osteotomy (*P* = 0.0075) and arthrodesis groups (*P* = 0.0004) than in the control group. In the PL area of the first metatarsal, significantly lower HU ratios were observed in the arthrodesis group than in the control group (*P* = 0.0034). No significant difference in the HU ratios could be detected in the PM and PL areas of the medial cuneiform (*P* > 0.05) and the DM, DL, and PM areas in the first metatarsal (*P* > 0.05) among the three groups (Fig. 2).

**Fig. 3:**
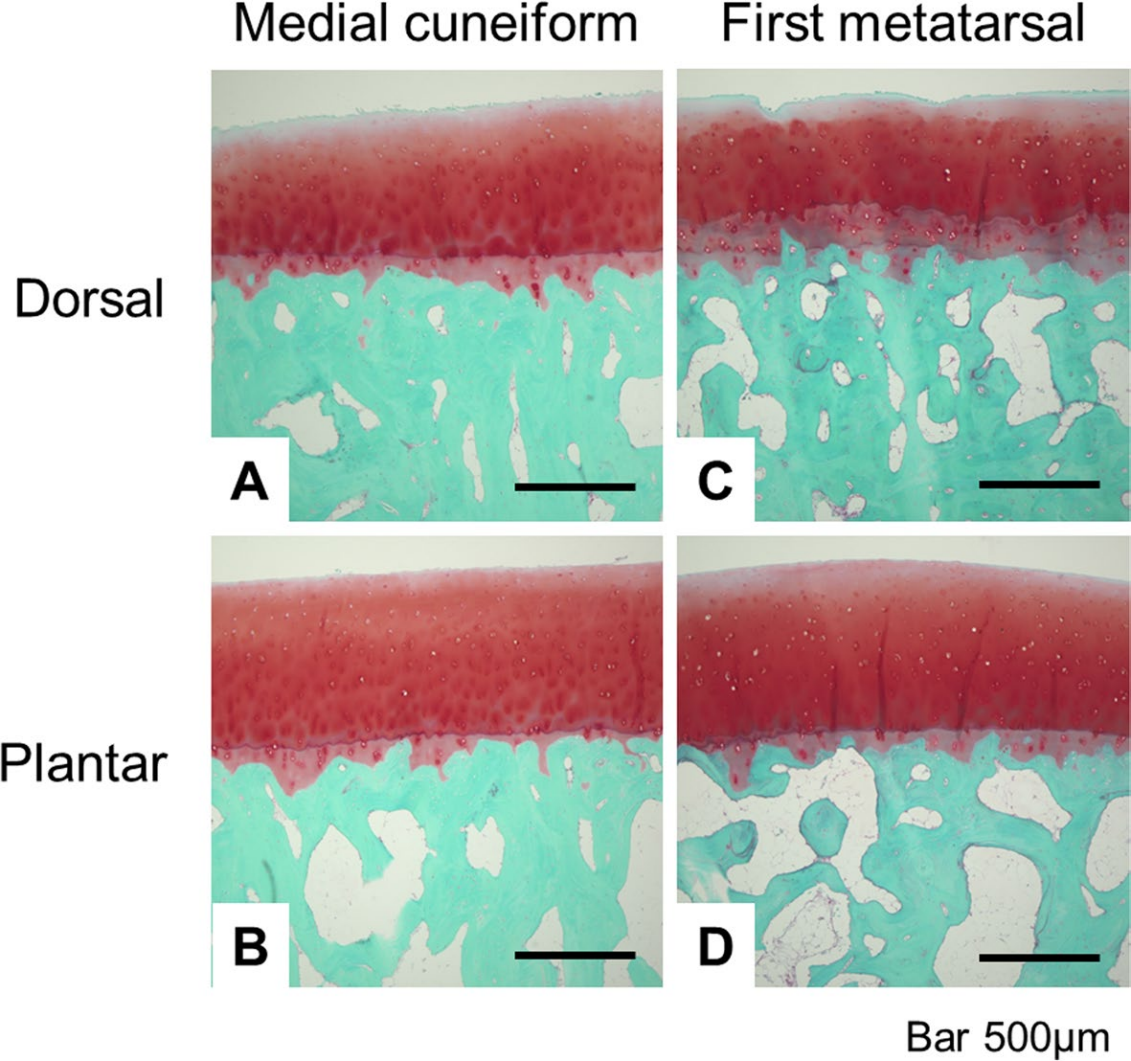
Representative histological images of the medial cuneiform (**A**, **B**) and the first metatarsal (**C**, **D**) in the arthrodesis group. **A**, **C**: Slight degenerative changes of the articular cartilage and increased bone volume fraction in the dorsal region. **B**, **D**: Normal articular cartilage in the plantar region

On histology, the OARSI scoring system indicated close to normal articular cartilage scores for all the subdivisions in both the medial cuneiform and the first metatarsal (Table 3 and Fig. 3). Furthermore, no significant difference could be detected in the OARSI scores of the medial cuneiform (*P* = 0.83) and the first metatarsal (*P* = 0.45) between patients with and without rheumatoid arthritis.

**Table 3:** Histopathological grade of the articular cartilage of the medial cuneiform and first metatarsal in the arthrodesis group

	**Dorsomedial**	**Dorsolateral**	**Plantomedial**	**Plantolateral**	*P* value
Medial cuneiform	1.26 (0.87–1.66)	1.00 (0.61–1.39)	0.74 (0.27–1.21)	0.74 (0.34–1.14)	0.21
First metatarsal	1.09 (0.72–1.45)	1.13 (0.8–1.46)	0.83 (0.55–1.11)	0.74 (0.44–1.04)	0.21

All parameters were statistically analyzed using one-way ANOVA. Each parameter is shown as the mean (95% confidence interval)

## Discussion

This study investigated the distributional patterns of subchondral bone density in the first TMT joint of those with HV and the associated histopathological features seen in severe HV and compared it to those with normal feet. Interestingly, the arthrodesis group (severe HV) demonstrated significantly higher HU ratios in the DM area of the medial cuneiform, and lower HU ratios in the PL area of the first metatarsal. Additionally, lower HU ratios were identified in the DL area of the medial cuneiform in both the osteotomy and the arthrodesis group. The most important finding in this study is that the histopathological evaluation revealed normal or mild degenerative changes in the articular cartilage of the first TMT joint, both in the medial cuneiform and in the first metatarsal, despite the presence of severe metatarsus primus varus in the arthrodesis group.

Subchondral bone density has been investigated in several joints, such as the hip, knee, shoulder [18], and ankle [14]; however, only few cadaveric studies have evaluated the distribution of subchondral bone density in the first TMT joint [4, 19]. Maximum density areas were frequently observed along the medial edge in both the medial cuneiform and the first metatarsal. Additionally, it was observed that force transmission did not occur through the center of the first TMT joint in the elderly without HV [19]. The distributional patterns of the subchondral bone density in the subdivided areas are associated with the mechanical stress borne by the first TMT joint. A low HU value indicates osteopenia and osteoporosis [13, 20], as well as decreased mechanical stress on the joints [21]. In the first TMT joint of patients with HV, dorsiflexion, inversion, and adduction were observed during weight-bearing when compared with that in those with normal feet [22, 23]. Low subchondral bone density of the PL area of the first metatarsal and the DL area of the medial cuneiform is therefore induced by decreased stress on them due to adduction and dorsiflexion of the first metatarsal in severe HV. The peroneus longus tendon attaches to the plantar lateral base of the first metatarsal by a strong band [24]. Thus, these distributional changes might have been initially induced by the dysfunction of the peroneus longus tendon. Subsequently, increased stress could cause high subchondral bone density in the DM area of the medial cuneiform receiving long-term mechanical stress in severe HV with metatarsus primus varus.

This is the first study to investigate the histopathological findings in the osteochondral unit of the first TMT joint in severe HV. Subchondral bone plate changes are related to articular cartilage degeneration responsible for the progression of OA [25]. Although subchondral bone density in the DM area of the medial cuneiform was higher in patients with severe HV, nearly normal articular cartilage was observed in the same region through histopathological examination. A cadaveric study reported that the bone mineral density of the first metatarsal was higher in the metatarsal head region than in the metatarsal base [26]. These findings were explained by the relatively lower mechanical stress in the normal midfoot than in the forefoot. Biomechanically, the relative contact area of the first TMT joint varied with the ankle position; on eversion, this area decreased significantly [27]. Among the weight-bearing joints, the first TMT joint receives relatively low mechanical stress which is reduced further by the altered rearfoot alignment of HV on eversion. The articular cartilage of the first TMT joint is therefore maintained, regardless of the severity of metatarsus primus varus. A better understanding of the distribution of the subchondral bone density and histopathological findings of the first TMT joint will aid future research focusing on the foot biomechanics with respect to the first TMT joint. This may assist in the clinical decision-making for surgical procedures in patients with hallux valgus.

Lapidus arthrodesis can provide efficient correction of the deformity and stabilization of the first ray, and good clinical outcomes have been reported in patients with moderate to severe HV [28]. A significant decrease in the pressure under the second metatarsal head and increased pressure under the fifth metatarsal head were observed after Lapidus arthrodesis, suggesting that it has a positive effect on promoting forefoot load sharing [29]; still, the procedure is technically demanding and is associated with a relatively high complication rate [30]. The mobility of the first TMT joint plays a key role in handling forced overload emanating from the high load of the second and third TMT joints [27]. The risk of adversely affecting the adjacent joint in terms of mechanical loading is a particular concern in Lapidus arthrodesis. Finite element analysis demonstrated that the first and second TMT joint fusion resulted in biomechanical performance changes in the foot and ankle complex, while the contact pressure of the naviculocuneiform and fifth metatarsal-cuboid joints increased by 27% and 40% at the terminal stance of gait, respectively; long-term consequences of these changes may therefore include arthritis of these joints [31]. In the present study, although HV exhibited severe deformity with an excessive IMA, nearly normal cartilage was histologically found in the first TMT joint. Additionally, the first ray hypermobility was improved by proximal metatarsal osteotomy combined with a distal soft tissue procedure, without the need for arthrodesis of the TMT joint [30, 32]. Recently, Lapidus arthrodesis has been employed for the correction of moderate to severe HV with metatarsus primus varus, HV with hypermobility of the first ray, HV in adolescents with generalized ligamentous laxity, and recurrence of HV after surgery [33]. However, the indications for Lapidus arthrodesis should be reconsidered in OA of the first TMT; it could be limited to patients with severe HV with degradation of the first TMT joint, or very elderly patients with decreased physical activity in whom the adjacent joints would be less affected by the first TMT arthrodesis.

This study has limitations. Patients with foot or ankle problems who underwent CT scans other than healthy participants without HV were enrolled in the control group. Consequently, a small number of younger patients were included as the controls. In addition, the gender difference of the participants that can affect subchondral density was identified among the three groups. Age- and gender-matched and healthy individuals without HV should be investigated as controls to delineate the changes in the distributional pattern of subchondral bone density and histopathological features in young individuals with normal feet to elderly patients with hallux valgus in the future.

## Conclusions

Although high subchondral bone density was identified in the DM area of the medial cuneiform in severe HV with metatarsus primus varus, only mild degradation was observed in the articular cartilage of the first TMT joint on histology. Our findings suggest that the indications for first TMT arthrodesis should be reconsidered based on the severity of the osteoarthritic changes in the first TMT joint.

## Data Availability

The datasets used and analyzed during the current study are available from the corresponding author on reasonable request.

## Abbreviations

HV: Hallux valgus
TMT: Tarsometatarsal
OA: Osteoarthritis
HR-pQCT: High-resolution peripheral quantitative computed tomography
CT: Computed tomography
HU: Hounsfield unit
HVA: Hallux valgus angle
IMAs: Intermetatarsal angles
DM: Dorsomedial
DL: Dorsolateral
PM: Plantomedial
PL: Plantolateral
OARSI: Osteoarthritis Research Society International
ANOVA: Analysis of variance
